# Genetically modified and unmodified cellular approaches to enhance graft versus leukemia effect, without increasing graft versus host disease: the use of allogeneic cytokine-induced killer cells

**DOI:** 10.3389/fimmu.2024.1459175

**Published:** 2024-10-24

**Authors:** Benedetta Rambaldi, Giuliana Rizzuto, Alessandro Rambaldi, Martino Introna

**Affiliations:** ^1^ Dipartimento di Oncologia ed Ematologia, Ospedale Papa Giovanni XXIII, Bergamo, Italy; ^2^ Molecular and Translational Medicine Doctoral Program (DIMET), University of Milano-Bicocca, Monza, Italy; ^3^ Department of Oncology and Hematology, Università degli Studi di Milano, Milan, Italy

**Keywords:** hematopoietic cell transplant, donor lymphocyte infusion (DLI), cytokine-induced killer (CIK) cells, leukemia, graft versus leukemia (GVL), chimeric antigen receptor (CAR)

## Abstract

Although allogeneic hematopoietic cell transplantation (HCT) represents a curative approach for many patients with hematological diseases, post-transplantation relapse occurs in 20-50% of cases, representing the primary cause of treatment failure and mortality. Alloreactive donor T cells are responsible for the graft versus leukemia (GvL) effect, which represents the key mechanism for the long-term curative effect of HCT. However, the downside is represented by graft versus host disease (GvHD), largely contributing to transplant-related mortality (TRM). Multiple factors play a role in regulating the delicate balance between GvL and GvHD, such as the optimization of the donor HLA and KIR match, the type of graft source, and the adaptive use of post-transplant cellular therapy. In addition to the standard donor lymphocyte infusion (DLI), several attempts were made to favor the GvL effect without increasing the GvHD risk. Selected DLI, NK DLI, activated DLI and more sophisticated genetically engineered cells can be employed. In this scenario, cytokine-induced killer (CIK) cells represent a suitable tool to boost GvL while minimizing GvHD. CIK cells are T lymphocytes activated in culture in the presence of monoclonal antibodies against CD3 (OKT3), interferon-gamma (IFN-g), and interleukin-2 (IL-2), characterized by the expression of markers typical of NK cells and T cells (CD3^+^, CD56^+^, with a prevalent CD8^+^ phenotype). CIK cells can mediate cytotoxicity through both MHC and non-MHC restricted recognition, which is the so‐called “dual‐functional capability” and display minimum alloreactivity. Allogeneic CIK cells showed a favorable rate of response, especially in the setting of minimal residual disease, with a rate of GvHD not exceeding 25%. Finally, the CIK cell platform can be adapted for chimeric antigen receptor (CAR) cell strategy, showing promising results in both preclinical and clinical settings. In this review, we describe the main immunological basis for the development of the GvL and the possible cellular therapy approaches used to boost it, with a particular focus on the use of CIK cells.

## 1 Introduction

Allogeneic hematopoietic cell transplantation (HCT) remains a curative approach for many patients with malignant and non-malignant hematological diseases (1). Acute myeloid leukemia (AML) represents the most frequent indication for HCT, accounting for 38% of transplants in Europe (2). In medically fit patients, HCT represents the most used post-remission therapy, reducing relapse incidence by eliminating residual leukemic cells (3). However, post-transplantation relapse occurs in 20-50% of patients, still representing the primary cause of treatment failure and mortality (4–6).

The anti-leukemic effect of transplant is due to the conditioning regimen, consisting of high-dose chemotherapy with or without total body irradiation (TBI), and to the donor immune surveillance. The conditioning regimen aims not only to kill residual leukemic cells but also to reduce recipient bone marrow hematopoietic and immune cells. The intensity of the conditioning used depends on the fitness and age of the patient (7, 8). Although myeloablative conditioning (MAC) showed better activity in controlling the disease recurrence in AML and in myelodysplastic syndrome (MDS) patients, compared to reduced intensity conditioning (RIC) (9), the use of RIC regimens increased over time, allowing the treatment of elderly or unfit patients too.

Alloreactive donor T cells are responsible for the graft versus leukemia (GvL) effect, which represent the key mechanism for the long-term curative effect of HCT, especially after RIC HCT (10). However, donor T cells are also responsible for alloreactivity against normal tissues, leading to graft versus host disease (GvHD), largely contributing to the transplant-related mortality (TRM) (6). The first description of the GvL effect goes back to the experimental mouse model, in which the animals injected with leukemic cells were treated with TBI followed by the administration of isologous or homologous myeloid tissue. Both graft sources could restore the hematopoietic compartment, but only the latter one displayed the ability to protect from leukemia. However, it was also associated to lethal toxicity (diarrhea) (11). These preliminary experiments showed for the first time the tight connection between GvL and GvHD. In 1994, Gale and colleagues reproduced these data in patients, showing that HCT from sibling donors can potentially cure patients with AML, while a minor effect was seen using a syngeneic donor (12, 13).

Post-transplant relapse is often driven by the ability of the tumor to escape from the immunological surveillance, hampering the GvL activity (14). Principal mechanisms of immune escape after HCT are loss of mismatched human leukocyte antigen (HLA) haplotype (15–17) HLA downregulation (18–20), and inhibition of allogeneic T cells through overexpression of inhibitory receptors or the perturbation of anti- and pro-inflammatory cytokines (19). Moreover, different strategies are used to modulate the donor T cell alloreactivity and prevent GvHD, such as the optimization of the donor match, the T cell depletion (both *in vivo* and ex vivo), and several immunosuppressive drugs (21). However, the same approaches could hamper the desired GvL activity, increasing the risk of disease relapse. For these reasons, multiple strategies were tested to boost the GvL activity after transplant, including different cellular therapy approaches, such as donor lymphocyte infusion (DLI), selected DLI, activated DLI, cytokine-induced memory-like (CIML) NK cells, and cytokine-induced killer (CIK) cells. Finally, genetically modified lymphocytes using chimeric antigen receptor (CAR), represent a potent tool to boost the GvL in specific disease setting, where a target antigen is available.

This review aims to describe the main immunological basis for the development of the GvL after HCT and the possible cellular therapy approaches used to boost it, with a particular focus on the use of CIK cells.

## 2 Immunological basis of GvL: donor selection through HLA, KIR matching and graft source

**Figure 1:**
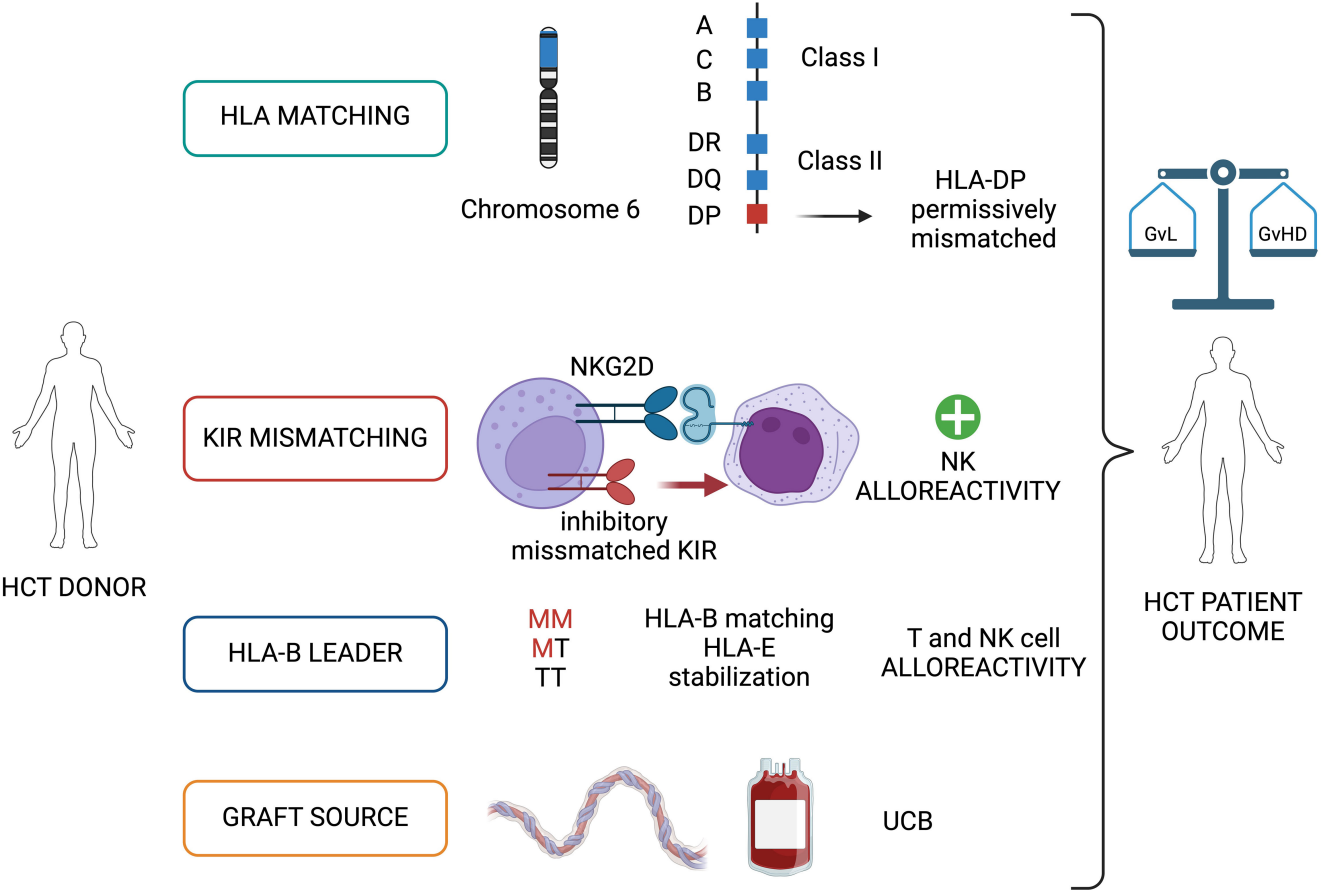
Graft versus Leukemia (GvL) immunological basis: donor selection through HLA, KIR matching and graft source. The donor-recipient matching for HLA and KIR, and the type of graft source (UCB) are detrimental factors during the selection of the HCT donor and contribute to the final balance between a GvL and GvHD. HCT, allogeneic hematopoietic cell transplantation; KIR, killer Ig-like receptor; UCB, umbilical cord blood; M, methionine; T, threonine; GvHD, graft versus host disease. Image created with BioRender.com.

The GvL activity can be predicted and modulated during the selection of the suitable transplant donor, in terms of HLA matching, killer Ig-like receptor (KIR) alloreactivity, and type of graft source (**Figure 1**).

### 2.1 HLA matching

The HLA genes, which are located on the short arm of chromosome 6 (6p21.3), encode for the molecules responsible for presenting antigens to T lymphocytes, making these genes the key players of the alloreactivity. The HLA loci are divided into two subclasses: HLA class I and HLA class II. Generally, HLA class I alleles -A, -B, and -C are expressed on all nucleated cells and display antigen to CD8^+^ T cells, while HLA class II alleles -DR, -DQ, -DP are expressed on antigen-presenting cells (APCs) and initiate a response by CD4^+^ T cells, playing an important role in recognizing non-self, infected or malignant cells. In the thymus, T cells with various T cell receptors (TCRs) undergo positive selection and negative selection, among which those that do not respond to the HLA-antigen peptide complex or have a strong response to self-antigens are eliminated (22). In addition to classic HLA class I and HLA class II alleles, there are also fewer polymorphic genes in these regions, including HLA-E, HLA-F, and HLA-G in HLA class I regions and HLA-DO and HLA-DM in HLA class II regions. These less polymorphic genes are also important regulators of the immune system. To date, more than 30,000 HLA alleles have been identified (23).

Considering the role of HLA proteins in the discrimination between the self and non-self, the search for an HLA-matched donor is crucial in the setting of tissue and organ transplantation, especially for HCT (24). HLA-matched related donors (MRD) generally share both alleles of HLA-A, -B, -C, -DR, -DQ, and -DP (12 of 12) because they have inherited the same parental copies of chromosome 6 encompassing the major histocompatibility complex (MHC). In MRD HCT the targets of T-cell alloreactivity are almost exclusively minor histocompatibility antigens (mHAgs), mediated by donor naïve T cells. This explains the lower rate of GvHD but also a less potent GvL effect in MRD HCT compared to transplants performed with alternative donors (25). In HLA-matched unrelated donor (MUD) HCT, patients and their HLA-matched donors carry the same 8 of 8 alleles at HLA-A, -B, -C, and -DRB1, without sharing their respective genetic backgrounds. Genetic disparity between unrelated individuals frequently includes the HLA-DRB3/4/5, -DQ, and -DP loci which are targets of direct T-cell alloreactivity. In this case, both naïve and memory donor T cells can recognize different recipient HLA-restricted peptides regardless of their derivation from novel or recall antigens. Due to strong linkage disequilibrium between HLA-DRB1 and -DRB3/4/5 and -DQ, mismatches for these genes are relatively rare in MUD. In contrast, HLA-DP mismatches are present in over 80% of MUD HCT, representing a key aspect of both GvL and GvHD in this transplant setting (26, 27). Moreover, HLA-DR^+^ leukemias and lymphomas generally co-express HLA-DP, making it an attractive GvL target (28). Of note, homozygosity for HLA-DP genes represents a frequent event, often resulting in unidirectional (only graft-versus-host [GvH] or host-versus-graft [HvG] direction) HLA-DP mismatches in MUD-HCT. Indeed, HLA-DPB1 mismatches are associated with increased risks of acute GvHD after 8 of 8 HLA-matched MUD HCT, but also with a significantly reduced risk of leukemia relapse, resulting in no net impact on overall survival (OS) (25). The notion of permissive mismatches is based on accumulating evidence suggesting that limited alloreactivity is sufficient for GvL, whereas aggressive alloreactivity can lead to clinically uncontrollable GvHD (29). In this view, permissive mismatches are those eliciting limited alloreactivity, shifting the balance from GvHD to GvL (30, 31). Current guidelines for patients transplanted for malignant disease recommend selection of an HLA-DPB1 allele-matched or permissively mismatched MUD, available with a probability of at least 70% (32). Also, Rutten and colleagues, in ex vivo analysis, first demonstrated the importance of CD4^+^ T cells alloreactive to HLA-DP in patients responding to DLI after MUD HCT (33). Moreover, to enhance the GvL effect, some groups propose to use *in vitro* expanded cytolytic T-lymphocytes (CTL) selected from the CD4^+^ naïve T cells alloreactive against HLA-DP specificities (34).

In the absence of an HLA-matched donor, the use of an alternative donor, such as an HLA-mismatched unrelated donor (MMUD, less than 8/8 alleles at HLA-A, -B, -C, and -DRB1), umbilical cord blood (UCB) or haploidentical related donor, has increased the number of patients that can access to the transplant procedure. However, in the MMUD setting, even a single allele mismatch negatively impacts patient outcomes after transplant (35). Specific strategies are needed to achieve the attenuation of the donor T cell alloreactivity in haploidentical HCT. The use of high-dose post-transplant cyclophosphamide (PTCy) has become the most frequently used due to its relatively easy applicability (36). Other strategies are represented by the use of rabbit-derived anti-thymoglobulin polyclonal antibodies (REFS) granulocyte-colony stimulating factor (G-CSF) mobilized bone marrow grafts with extensive immunosuppression (37) or ex vivo graft manipulation, such as CD34 megadose (38, 39) or a/b T cell depletion (40). Finally, the Perugia group proposed a novel approach using a T-cell-depleted graft and a subsequent infusion of regulatory and conventional T cells, without any post-transplant immune suppression (41).

Altogether, the GvL effect can benefit from a careful choice of the HLA-compatible donor, particularly in the setting of permissive HLA-DP mismatched MUD (**Figure 1**).

### 2.2 NK cell alloreactivity and KIR matching

A special emphasis is being placed on using natural killer (NK) cells to harness both innate and adaptive immunity after HCT (42). NK cells are uniquely regulated by activating and inhibitory receptors and can mediate a critical GvL effect, also referred to as NK cell alloreactivity, without mediating GvHD (43–46). NK cell alloreactivity can potentially provide a better antitumor effect, as documented by lower relapse rates and better survival in patients with higher NK cell numbers early post-transplant (47, 48). Cytotoxic activity of NK cells is mediated primarily by a balance between inhibitory and activating receptors expressed on the cell surface, the former being mainly accounted KIRs that recognize HLA class I molecules on the surface of target cells (49). When NK cells encounter the matching HLA-class I ligand for their inhibitory KIR, they are considered “educated” or “licensed” and refrain from an attack on healthy tissues under a steady state. When NK cells are accustomed to this inhibitory signal and subsequently encounter a cell that does not express the appropriate KIR-ligand (“missing ligand”), they can mount an effector response, if the target also expresses stress-ligands that trigger activating NK-cell receptors (such as natural killer group 2 member D, NKG2D, **Figure 1**). Due to the lack of exposure to their corresponding ligand, unlicensed NK cells are “un-educated” and hyporesponsive at a steady state rather than being triggered by self-tissues lacking the ligand (50). Several models of donor-recipient NK cell alloreactivity have been proposed. The KIR ligand incompatibility (*ligand–ligand*) model, in which NK cells will react and kill host cells that lack the HLA class I ligand(s) for inhibitory KIR, was first proposed by the Perugia group (51). Alloreactive NK cells in the GvH direction helped promote engraftment and GvL, resulting in a reduced risk of leukemia relapse and better survival in adults with AML without increasing the rate of GvHD (52). An alternative model called the *receptor-ligand* or *missing-ligand* model, proposed that NK cells will react if at least one KIR gene expressed in the donor’s NK cell repertoire does not recognize any of the HLA molecules in the recipient’s ligand repertoire. This model showed a better ability to predict NK alloreactivity and lower the risk of leukemia relapse in a pediatric study of patients with high-risk leukemia given CD34^+^ selected haploidentical graft (53). The *educational/missing licensing proof model* required that the NK cells were educated against a specific antigen in order to become alloreactive when encounter a recipient cell that lack its cognate ligand (54). Other groups have used the *KIR-haplotype model* which takes into consideration the presence or absence of a B-KIR haplotype in the donor, as a measure of enrichment for activating versus inhibitory KIRs. The use of this model demonstrated a reduced risk of leukemia relapse when patients were transplanted from donors with centromeric B-haplotypes (55, 56). Finally, the *KIR genotyping model*, analyzes KIR genes between donor and recipient to observe a correlation between KIR match/mismatch and transplant outcomes. This model has shown discrepant results in studies with different HCT platforms (57).

Although a definitive and easy-to-apply model for the evaluation of donor-recipient KIR alloreactivity is not currently available in clinical practice, the presence of a KIR mismatched have shown to positive impact the prognosis of leukemia patients after transplant, augmenting the NK-mediated GvL.

### 2.3 T and NK cells alloreactivity through the HLA-B leader

The last factor to take into consideration for modulating T and NK cell alloreactivity is the HLA-B leader (**Figure 1**). The leader sequence refers to the peptide encoded by exon 1 of the 7 exons that comprise the HLA-B gene. It has been shown that in HLA-B, the leader sequence encodes methionine (M) or threonine (T) at position -21 and can give rise to TT, MT, or MM genotypes. In the setting of MMUD, there is an increased risk of severe acute GvHD when the patient has an M in the leader sequence and when the leader sequence is mismatched (58). Moreover, the leader sequence of HLA class-I molecules is presented by the non-classical class I molecule, HLA-E, and stabilizes HLA-E expression on the cell surface, enhancing binding to receptors on NK cells. It is hypothesized that HLA-B presentation by HLA-E and subsequent recognition by NK cells may contribute to improvements in non-relapse mortality (NRM) that have been demonstrated in several HCT settings (MMUD, haploidentical, and UCB HCT) (59–61).

The HLA-B leader status contributes regulating the GvL/GvHD balance and may inform relapse and NRM risk after HCT.

### 2.4 Graft source and alloreactivity: the case of UCB transplant

Multiple data are now favoring the use of UCB HCT for patients with high-risk leukemia, suggesting the role of the graft source in determining the GvL effect (**Figure 1**). In the retrospective analysis performed by Milano and colleagues, 582 consecutive patients with acute leukemia or myelodysplastic syndrome received a first myeloablative hematopoietic-cell transplant from an UCB (140 patients), an MUD, or an MMUD (344 and 98 patients, respectively). Authors observed that among patients with pre-transplant minimal residual disease, the probability of OS after receipt of a transplant from a UCB was at least as favorable as that after receipt of a transplant from an MUD and was significantly higher than the probability after receipt of a transplant from an MMUD. Furthermore, the probability of relapse was lower in the UCB group than in either of the other groups, suggesting a higher GvL activity in the UCB group (62). In addition, the pediatric study by Verneris and colleagues revealed an enhanced GvL effect in acute leukemia patients after transplantation with 2 partially HLA-matched UCB units compared to 1 single unit UCB (63). In another prospective, multicenter, pediatric study recently published, 367 patients affected by AML/MDS undergoing T-cell replete HCT were analyzed. One-hundred and twelve patients underwent a UCB HCT, the remaining 255 received other cell sources. Although a higher incidence of poor prognosis features in the UCB group, these patients showed an unexpectedly favorable EFS (64.1%). In a multivariable analysis, the UCB cohort had significantly improved EFS, time to relapse, and reduced chronic GvHD, with some evidence of improved OS. The effect appeared similar regardless of the minimal residual disease (MRD) status, suggesting that UCB HCT without serotherapy may be the optimal transplant option for children with myeloid malignancy (64). Finally, so far, there are no reports of HLA loss, as a strategy of leukemia immune escape, in the setting of UCB transplantation (Prof Luca Vago, personal communication).

UCB represents a suitable graft source to boost GvL after HCT and should be considered in the setting of high-risk disease.

### 2.5 Graft manipulation and alloreactivity

Different approaches were used to modify the graft in order to reduce the incidence of GvHD while maintaining the GvL effect (**Table 1**). The first strategy used the CD34^+^ megadose in the setting of haploidentical transplantation, without the addition of any other immunosuppression (38). This strategy showed low incidence of relapse and remarkably low incidence of GvHD. Another approach includes the depletion of possible alloreactive T cells, defined as α/β TCR positive cells, in association with ATG and rituximab. This strategy was mostly developed in the pediatric haploidentical setting by Locatelli and colleagues with promising results (40). Again, the group of Perugia further expanded the concept of CD34^+^ megadose, with the Treg/Tcon graft strategy, where a known dose of haploidentical regulatory (Treg) and conventional (Tcon) T cells were administrated to the patient. This strategy showed remarkably low incidence of both acute and chronic GvHD with a favorable relapse incidence (65, 66).

**Table 1:** Selection of main clinical trials on prophylactic graft manipulation strategies to prevent GvHD and enhance GvL.

Graft manipulation type	HCT setting	Patients (N)	GvHD prophy	aGVHD incidence	cGVHD incidence	Relapse incidence	Median FU months (range)	Reference
CD34^+^ megadose	Haplo	104	None	8/100 (8%)	5/70 (7%)	26/104 (25%)	22 (1-65)	Aversa et al. JCO 2005 (38)
a/b T and B cell depletion	Haplo	80	ATG Rituximab	24/80 (30%)	4/73 (5%)	19/80 (24%)	46 (26-60)	Locatelli et al. Blood 2017 (40)
Tregs/Tcons CD34^+^	Haplo	43	None	6/41 (15%)	1/41 (2%)	2/41 (5%)	46 (18-65)	Martelli et al. Blood 2014 (65)
Tregs/Tcons CD34^+^	Haplo	50	None	15/50 (30%)	1/50 (2%)	2/50 (4%)	34 (5-72)	Pierini et al. Blood Adv 2021 (66)
CD45RA^+^ depletion	MRD	35	Tac	23/35 (66%)	3/35 (9%)	21% at 2yrs	/	Bleakley et al. JCI 2015 (67)
CD45RA^+^ depletion	MRD/MUD	138	Tac +/- Mtx and MMF	103/138 (75%)	9/138 (7%)	23% at 3yrs	49 (9-61)	Bleakley et al. JCO 2022 (68)

N, number; prophy, prophylaxis; MRD, matched related donor; MUD, matched unrelated donor; Haplo, haploidentical donor; yrs, year; aGvHD and cGvHD, acute and chronic graft versus host disease; Treg, regulatory T cells; Tcon, conventional T cells; Tac, tacrolimus; Mtx, methotrexate; MMF, mycophenolate mofetil.

Finally, Bleakley and colleagues, elegantly tested the effect of a naïve T cell depleted graft in the setting of MRD or MUD, by eliminating the CD45RA^+^ cells from the graft. These trials showed a non-detrimental impact on relapse incidence with low cGvHD, but an aGvHD still above 50% (67, 68).

## 3 Cellular therapies to boost GvL effect

**Figure 2:**
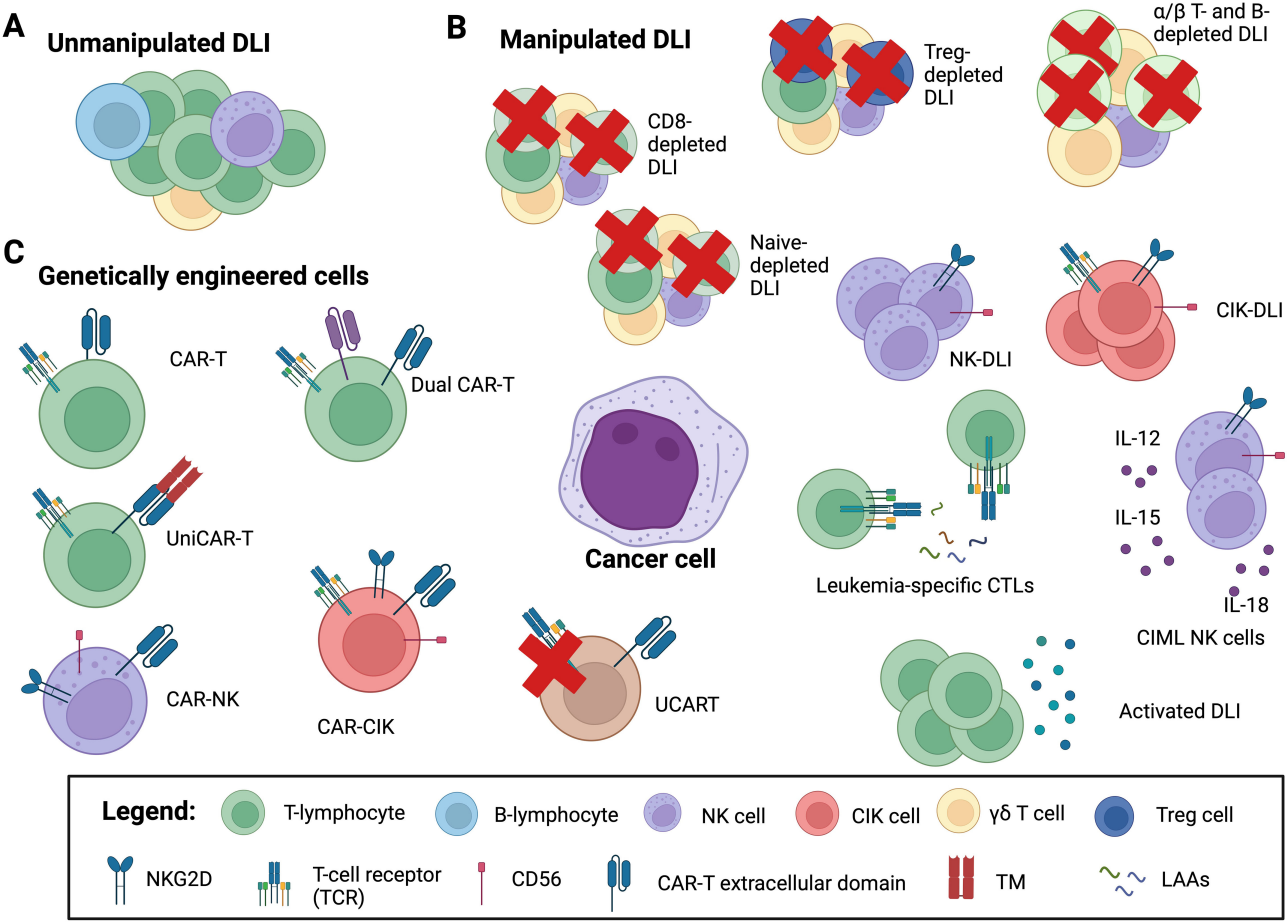
Adoptive cellular therapy in the post-HCT setting. Adoptive cellular therapy strategies include: **(A)** unmanipulated donor lymphocyte infusions (DLI); **(B)** manipulated DLI: CD8^+^ T cell depleted DLI, α/β T and B cell depleted DLI and CD45RA^+^ naïve T cell depleted DLI to mitigate the risk of graft versus host disease (GvHD); regulatory T cell (Treg) depleted DLI, to enhance graft versus leukemia (GvL) activity; natural killer (NK) cell and cytokine-induced killer cell (CIK) DLI to both minimize GvHD and increase GvL; cytokine-induced memory-like (CIML) NK cell DLI for enhancing NK killing capacity and persistence; activated DLI, either with cytokine (IL-2, IL-15, IL-21) or with dendritic cells or G-CSF primed DLI to augment graft versus leukemia (GvL) activity; leukemia-specific CTLs (cytotoxic T lymphocytes) and leukemia-associated antigens (LAAs) to redirect specificity; **(C)** genetically engineer cells: chimeric antigen receptor (CAR) T cells, genetically engineered to recognize and kill targets: allogeneic CAR-T; “universal” CAR (UniCAR) cells are inactive under physiologic conditions, but can recognize a soluble adaptor, called targeting module (TM), directed against the specific target; CAR-NK and CAR-CIK cells, to minimize the risk of GvHD; dual-CAR-T target multiple antigens simultaneously to circumvent immune escape arising from antigen-negative disease; UCART where the endogenous TCR can be disrupted in mature T cells to abrogate GvHD potential. Image created with BioRender.com.

Adaptive and innate immunity are both crucial in maintaining the delicate balance between GvHD and GvL effect. The major players in this scenario are the T and NK cells lymphocytes. Different strategies were tested to use unmodified or modified T and NK cells in the setting of HCT to prevent or treat leukemia recurrence (**Figure 2**). Here we describe the major utilized cellular therapy approaches, highlining the ability of CIK cells to combine both T and NK cell properties.

### 3.1 Secondary HCT

Although outcomes for AML/MDS patients relapsed after HCT are quite disappointing, a second HCT may offer a chance of survival in selected patients, representing a valid cellular therapy option. However, a second HCT is generally limited to medically fit patients due a high rate of TRM, reaching up to 73.5% in the first series of studies (69). A more encouraging TRM was reported with the introduction of RIC regimens, ranging from 30% to zero, mostly associated with a short interval between the two transplants and the disease status at the time of the second HCT (70). A recent retrospective single-center study analyzed data from 407 consecutive patients with relapsed AML/MDS after the first HCT. Sixty-two patients had a second HCT (15%) and 345 did not. The 2-year cumulative incidence rates of NRM and relapse following the second HCT were 26% and 50%, respectively. Patients who underwent a second HCT had a better outcome, compared to patients who did not, with 5-year OS rates of 25% and 7%, respectively. In this study, the use of a second HCT was associated with longer survival especially in patients with longer remission after the first HCT (71). Since the loss of mismatch, HLA is one of the most relevant and understood mechanisms of immune escape after HCT (15, 16, 72, 73), a careful choice of the second HCT donor should be mandatory (74). In addition, emerging data on the use of easily accessible, alternative donor sources, such as UCB or haploidentical donors, may promote the practice of switching donors for second allotransplants. Indeed, in patients who relapsed with an HLA loss, a second HCT using a donor with a different HLA haplotype provided promising results (16). Different retrospective studies have tried to determine which is the best donor for the second HCT, with different results. In one single center experience including 40 patients undergone a second HCT, authors showed that an allograft with a new mismatched haplotype may improve outcomes following a second HCT (75). However, in a retrospective German registry study on 179 patients undergone a second HCT, changing the stem cell donor for the second transplant did not provide a significant advantage for disease recurrence or long-term survival, compared with the choice of the original donor for the second transplant (76). Of note, no haploidentical or UCB transplants were included in this analysis. Moreover, in a retrospective multicenter EBMT study, including 556 patients who underwent a second HCT, similar outcomes were observed in patients after a second HCT with the same or different matched donor. In this analysis, patients were divided into three groups based on the type of the second HCT donor: same donor, different matched donor, or haploidentical donor. The two-year leukemia-free survival (LFS) rate was 23.5%, 23.7%, and 21.8%, respectively. In addition, a non-T-cell depleted haploidentical transplant was associated with a similar relapse rate, but higher NRM (77). A different study from the EBMT conducted in acute lymphoblastic leukemia (ALL) patients demonstrated that T-cell replete haploidentical donors in second HCT had a 1.5-fold higher 2-year OS (49% vs. 31%) albeit non statically difference in OS when choosing either a MUD or haploidentical donor (78). Finally, some groups showed the feasibility of a UCB for a second HCT. In a retrospective Japanese analysis of 263 consecutive patients with different hematological malignancies, OS, NRM, and relapse at 2-years were 16.7%, 46.9%, and 40.6%, respectively. In multivariate analysis, younger age (<50), good PS (0-1), and long interval between HCTs (≧1 year) showed a superior OS (79). Similar positive results were collected in a pediatric series treated with a second/third UCB HCT, showing 3-year OS and EFS of 69.2% and 64.9%, respectively. The cumulative incidence of TRM at 3 years was 19.2%. Notably, no toxicity-related deaths were seen in the 16 patients who received UCB after 2015, suggesting an improvement in transplant care in this setting. The 3-year cumulative incidence of relapse was 15.9%, remarkably low for these very high-risk patients (80). In our center, we retrospectively collected data from 61 patients undergone a second HCT. Forty-five patients received the second HCT for disease relapse, while 16 for graft failure. Seventy percent of the patients were conditioned using a reduced-intensity regimen. In 54% of the patients a different donor was chosen for the second HCT and in 6 patients a UCB was used. Concerning disease status at the second HCT, 36% were in CR and 64% had active disease. At a median follow-up of 8.36 months (range: 0.2 – 276.3), 18 patients were alive. Twenty-seven patients died due to disease recurrence or progression. The 5-year cumulative incidence of NRM was 22%. The 5-year OS and PFS rates were 29% and 28%, respectively. Patients undergoing second HCT due to graft failure exhibited a 56% survival rate, higher compared to ones transplanted for disease recurrence (20%) (81).

Overall, recent data have shown an acceptable NRM and reasonable long-term survival in selected patients who underwent a second HCT.

### 3.2 Donor lymphocyte infusion

DLI can induce a GvL effect, defined as the ability of donor T lymphocytes to recognize and kill leukemia cells. The efficacy and toxicity of a DLI are hard to predict and depend on the wide range of different cell populations present in a leukapheresis product. In the DLI product, the majority of lymphocytes consists of CD3^+^ T cells (75-80%) (α/β T cells, regulatory T cells, and gd T cells), followed by NK cells (5-20%) and B cells (5%) (82, 83). ab T cells represent the largest and best-studied effector population, mediating alloreactivity by recognizing minor histocompatibility antigens (MiHA). Unmodified DLIs are the simplest way to increase GvL reactivity after HCT (**Figure 2**). However, studies have given different results, due to multiple variables, such as the type of conditioning regimen, *in vivo*/ex vivo T-cell depletion, post-transplant immunosuppression, the source of DLI, (unstimulated leukapheresis or G-CSF-stimulated products), fresh or cryopreserved products, the interval between the HCT and the first DLI, cell doses, and the number of infusions (82, 84). From the beginning, DLI was successfully employed in chronic myeloid leukemia (CML) (85), while less impressive results were observed in patients affected by AML (86, 87).

DLI therapy can be classified based on treatment intention into:

1. therapeutic, when used for the treatment of hematological relapse, often performed in conjunction with another anti-leukemic treatment;
2. preemptive, when used for the treatment of minimal residual disease or mixed chimerism;
3. prophylactic, when used in patients in disease remission but with a high risk of relapse (82, 83).

DLI was also tested in combination with other anti-leukemic drugs for both relapse treatment or maintenance after HCT. Schroreder and colleagues analyzed 154 patients with AML and MDS who relapsed after HCT and were treated with 5-azacytidine (AZA). All patients received a median number of 4 courses of AZA of those, 105 patients received concomitant DLI. Complete and partial remission rates were observed in 27% and 6%, respectively. The OS at 2 years was 29%, in MDS patients the 2 years OS was 66% and correlated with disease burden. Therefore, the authors conclude that AZA and DLI is an effective and well-tolerated treatment option in R/R patients after HCT, particularly for those with low disease burden (88). Later, in 2021, Guillaume retrospectively analyzed a cohort of 77 AML and MDS patients considered at high risk based on either their genomic or clinical status at transplantation. Following allogeneic transplantation, they received at least 1 cycle of prophylactic or preemptive low-dose AZA with or without escalating doses of DLI. Almost one-half of the patients were able to receive the full 12 cycles of AZA, and a majority (79%) received at least one DLI. Among the whole cohort, 19 patients relapsed, with a cumulative incidence of relapse (CIR) was 22% at 24 months. OS and progression-free survival (PFS) at 24 months were 70.8% and 68.3%, respectively. Grade II-IV acute GvHD and chronic GvHD were 27.4% and 45%, respectively (89).

Finally, some studies compared the efficacy of DLI in treating disease relapse with a second HCT. In one retrospective study 89 patients with a relapse disease or a graft failure, were treated with a second HCT (56%) or DLI (44%). The same HCT donor of the first transplant was used in 50% of the patients. The median number of DLI administered was 2 (range 1-11). This study showed that a second HCT may improve outcomes when performed for relapse if patients achieve a CR at the time of the second HCT, while DLI may be reserved for patients with active disease (90). In a retrospective EBMT study involving 418 adults with relapsed AML who received a second HCT (N=137) or DLI (N=281) there was no difference in OS whether a second HCT or DLI was prescribed, with a 5-year OS of 19% and 15% for second HCT and DLI, respectively. The best outcomes seem to be achieved in patients relapsing after 6 months from the first HCT or those in complete remission at the time of either second HCT or DLI (91).

The most feared adverse event following DLI therapy is the onset of GvHD, mediated by the infusion of donor lymphocytes, not controlled by immunosuppressive therapy (92). Depending from the DLI source and from the study the incidence of aGvHD and cGvHD varies from 20-60% and 20-40%, respectively (82). For this reason, efforts have been made to develop more targeted cellular therapy strategies to promote the GvL effect without increasing the GvHD effect. **Table 2** outlines the major studies using modified or manipulated DLI divided according to the strategy used into different groups: selected T cell DLI, NK cell DLI, antigen-specific DLI and activated DLI. The first three groups include products of specific lymphocyte subset, such as Treg-depleted DLI, CD8^+^ αβ-depleted T cells, ab T cells against specific leukemic antigens (WT1, NPM1) and NK DLI, while the latter group includes products activated using different cytokine cocktails or drugs, such as CIK cells or cytokine-induced memory like (CIML) NK cells (**Figure 2**).

**Table 2:** Clinical trials on modified donor lymphocyte infusion (DLI): selected T cell, NK cell, antigen-specific, and activated DLI.

Study	DLI Type	Patients (N)	Setting	DLI donor source	Cell dose/N infusions	Response	GvHD	OS
Selected T cell DLI								
Meyer et al. Blood 2007 (219)	CD8-depleted DLI	23	Prophylaxis	MRD (3) MUD (13) MMUD (7)	DL1 1×10^6^ CD4^+^ T cells/kg (day +60 after MRD and +120 after MUD/MMUD) DL2 3×10^6^ DL3 1×10^7^ DL4 3×10^7^ CD4^+^ T cells/kg (day +60 to +90)	4/23 convert in full chimerism	5 transient grade I aGvHD, 2 (grade II/III) aGvHD	/
Orti et al. Transplantation 2009 (220)	CD8-depleted DLI	28	Treatment	MRD (12) MUD (11) MMUD (5)	MUD/MMUD 1×10^6^ CD4^+^ cells/kg MRD 3×10^6^ CD4^+^ cells/kg	ORR 5/11 Conversion to full donor chimerism	5/28 aGVHD (grade II-IV) 1 cGvHD	/
Maury et al. BJH 2014 (100)	CD25/regulatory T-cell-depleted DLI	17	Treatment	MRD (13) MUD (4)	1-10×10^7^ CD3^+^/kg	/	5 aGvHD 1 cGvHD	4 Alive in CR
Alyea et al. BMT 2004 (93) and Bachireddy et al. Blood 2014 (94)	CD8-depleted DLI	29	Treatment	MRD (28 MID (1))	Planned dose 3×10^7^ CD4^+^ cells/kg	7 CR	1 aGvHD (grade II) 1 cGvHD	/
Nikiforow et al. Hematological 2016 (101)	CD25/regulatory T-cell-depleted DLI	21	Prophylaxis	MRD (13) MUD (8)	DL1 1×10^7^ (n=6) DL2 3×10^7^ CD3^+^ cells/kg (n=15)	DL1 5 pts had PD, 1 SD. DL2 8 CR, 1 PR	1y GVHD 33%	1-y OS 53% (dose level 2)
Muffly et al. Blood Adv 2018 (96)	Donor-derived CD8+ memory T cell	15	Treatment	MRD	1×10^6^, 5×10^6^, or 10×10^6^ cells/Kg	ORR 67% (10/15): 7 CR, 1 PR, 2 SD	2 aGvHD (grade II)	Median OS 4.9 months
Dunaikina et al. BBMT 2021 (221)	Low-dose memory (CD45RA-depleted) DLI after αβ T cell-depleted haploidentical HCT	76/149	Pre-emptive	Haplo	25×10^3^/kg of CD3^+^ (day 0) 50×10^3^/kg of CD3^+^ (day +30, +60, +90, and +120)	2-year NRM 2%, CIR 25%, EFS 71%	grades II–IV aGVHD was 14% (vs 12% in the control group)	2-year relapse 25% EFS 71% OS 80%
Maung et al. BBMT 2021 (103)	Naïve-depleted DLI	16	Prophylaxis	MRD (8) MUD (8)	1×10^5^ CD3^+^/kg, 1×10^6^ CD3^+^/kg, 5×10^6^ CD3^+^/kg, 1×10^7^ CD3^+^/kg.	/	1 aGvHD (6.2%)(grade II) 1 cGvHD (6.2%)	2-y PFS 50% OS 68.8%
Castagna et al. Transplant and Cellular Therapy 2021 (222)	CD45RA+ Depleted DLI	23	Pre-emptive	Haplo	First dose: 5×10^5^ CD3^+^/kg, second dose: 1×10^6^ CD3^+^/kg, third dose: 5×10^6^ CD3^+^/kg.	/	1 aGvHD (grade II) 2 moderate cGvHD	1-y OS 79% 1-y PFS 75%; 100-days NRM 5%, 1-y NRM 12%
Vydra et al. Clinical Lymphoma, Myeloma and leukemia 2023 (223)	Allogeneic γ δ T Lymphocytes	7	Treatment	Haplo	1×10^6^ cells/kg (n = 3), 1×10^7^ cells/kg (n = 3), 1×10^8^ cells/kg (n = 1).	1 CR, 1 CRi, 1 SD, 1 NR	None	/
NK DLI								
Rizzieri et al. BBMT 2010 (224)	NK-DLI	30	Prophylaxis	MRD (16) Haplo (14)	Median NK cells: 10.6×10^6^ cells/kg (MRD) 9.21×10^6^ cells/kg (Haplo)	/	8 aGvHD (Grade I/IV) 1 cGvHD	1-yr OS 43% (MRD) 42% (Haplo)
Choi et al. BBMT 2014 (225)	NK-DLI	41	Prophylaxis	Haplo	DL1 0.2×10^8^ cells/kg (n = 3), DL2 0.5×10^8^ cells/kg (n = 3), DL3 1.0×10^8^ cells/kg (n = 8), DL4 ≥1.0×10^8^ cells/kg (n = 27), (at 2 and 3 weeks after HCT)	/	aGVHD (grade II/IV) 17% cGVHD (moderate to severe) 15%	Significant reduction in leukemia progression compared to control (74% vs 46%)
Shaffer et al. BBMT 2016 (226)	NK-DLI	8	Prophylaxis	Haplo	Median NK cells 10.6×10^6^/kg median CD3^+^ cells 2.1×10^3^/kg	CR: 2/8 pts; response, but relapse; 1/8: durable CR	None	Median OS 12.9 months
Choi et al. BBMT 2016 (227)	DNKI (donor natural killer cell)	51	Prophylaxis	Haplo	Median DNKI 0.5, 0.5, 1.0, and 2.0×10^8^/kg cells. (days +6, +9, +13, and +20 after HCT).	CR 1 month after HCT 57%	Grade 2 to 4 aGVHD: 28%, cGVHD 30%.	3-year CIR 75%
Jaiswal et al. Cytotherapy, 2017 (228)	CD56-enriched donor cell infusion	10	Prophylaxis	Haplo	minimum of 1×10^6^ CD56^+^ cells/Kg and <1×10^6^ CD3^+^CD56^−^ cells/Kg	/	0 aGvHD 2 cGVHD	5/10 relapsed. NRM 10%.
Antigen-specific DLI								
Chapuis et al. Sci Transl Med 2013 (104)	HLA-A*0201– restricted WT1-specific donor-derived CD8+ cytotoxic T cell (CTL)	11	Treatment Prophylaxis	MRD MUD	Maximum dose 10^10^ CTLs/m^2^	/	None	3 alive in CR
van Balen et al. Frontiers of immunology 2020 (229)	HA-1H T-Cell Receptor Gene Transfer to Redirect Virus-Specific T Cells	5/9	Treatment (1) and Prophylaxis (4)	MRD (2) MUD (3)	Not reported	No response in the treatment group	None	Two patients are alive and well without GVHD.
Lulla et al. Blood 2021 (230)	Leukemia-specific T cells (mLSTs) against PRAME, WT1, Survivin, and NY- ESO-1	25	Treatment and Pre-emptive	MRD (21) MUD (1) Haplo (3)	0.5 to 10×10^7^ cells/m^2^ for 6 infusions	1 CR and 1 PR (active disease cohort)	No aGVHD> grade 2	Not-yet-reached OS and LFS at 1.9 y
Activated DLI (both T and NK)								
Slavin et al. Blood 1996 (231)	IL-2-DLI	17	treatment	MRD	0.2×10^8^/kg to 4.6×10^8^/kg + rhIL-2	/	3 aGvHD (grade II-IV)	Median OS 38 months
Huang et al. JCI 2008 (232)	G-CSF-primed peripheral blood progenitor cells	33	Prophylaxis	MRD	1–2 × 10^8^ cells/Kg and 0.93×10^6^/Kg	15 relapses	6 aGvHD (Grade II/IV) 20 cGvHD	16 alive in CR
Yoon et al. BMT 2009 (233)	CD34 generated NK-DLI after *in vitro* expansion with IL-15 and IL-21	18	Prophylaxis	Haplo	Median 9.28×10^6^ cells/kg	No anti-leukemia effect	1 aGvHD 5 cGvHD	/
Hardy et al. Blood 2012 (234)	Tumor-derived donor lymphocyte (TDL)	8	Treatment	MRD (4) MUD (3) UBC (1)	Median 2.04×10^7^ TDL/kg	2 transient PET response and 2 mixed responses	None	/
Ho et al. Am J Hematol 2014 (235)	Dendritic cells (DC) + DLI	16	Treatment	MRD	DL1 5×10^6^ DC cells/kg DL2 1×10^7^ DC cells/kg DL3 5×10^7^ DC cells/kg 3×10^7^ CD3^+^cells/kg (1 day after DC)	durable remissions 4/14	1 aGVHD (Grade II) 1 cGVHD	/
Kottaridis et al. PLOS ONE 2015 (236)	Tumor-primed NK cells (TpNK)	7/13	Treatment	Haplo	DL1 1×10^6^ NK cells/kg DL2 5×10^6^ NK cells/kg DL3 1×10^7^ NK cells/kg.	At 6 mo: 3 patients in CR remain in CR, 1 in PR achieved CR1, 2 relapsed and one died	None	Median OS was 400 days post infusion
Jaiswal et al. BBMT 2016 (237)	G-CSF-primed DLI	31	Prophylaxis	Haplo	Days +35, 60, 90 (NMC) Days +21, 35, 60 (MAC)	10/10 PD (NMC)	No GvHD (NMC) aGvHD 31% (MAC) cGvHD 41.2 (MAC)	18-months OS 52.9% and PFS 43.9%
Ciurea S. et al. Blood 2017 (238)	mbIL21 expanded donor NK cells	13	Prophylaxis	Haplo	1×10^5^ to 1×10^8^ NK cells/kg (days -2, +7 and +28 after HCT)	/	aGvHD (grade I-II) 54%, none cGvHD	11/13 were alive with a median follow-up of 14.7 months.
Vela M. et al. Cancer letters 2018 (239)	IL-15/4-1BB-L activated NK	20 (10 previous)	Treatment	Haplo	DL1 5×10^7^ NKAE cells/kg, DL2 1×10^8^ NKAE cells/kg. + rhIL-2	CR/MRD^-^ 10 CR/MRD^+^ 6	None	4 patients alive with median FU of 750 days
Otegbeye et al. Transplantation and Cellular Therapy 2022 (240)	NK-DLI (donor-derived IL-2 ex vivo expanded)	3 (only 1 after HCT),	Treatment	Third-part non-matched healthy donor	DL1 1×10^7^/kg, DL2 2.5×10^7^/kg, DL3 5×10^7^/kg. (2 infusions in 2 weeks)	1/3 CRi, 1/3 SD with AML/MDS	None	/
Shapiro et al. JCI 2022 (120)	CIML-NK cells + rhIL-2	6	Treatment	Haplo	5-10×10^6^ cells/kg	3 CR 1 PR	None	/
Lee et al. Leukemia 2023 (241)	IL-15 and IL-21 activated donor NK cells (randomized study)	36/77	Prophylaxis	Haplo	1.0 × 10^8^/kg and 1.4 × 10^8^/kg (days +13 and +20)	/	No increased GvHD compared controls	Disease progression 35% vs 61%

N, number; MRD, matched related donor; MUD, matched unrelated donor; MMUD, mismatched unrelated donor; haplo, haploidentical donor; ORR, overall response rate; CR, complete response; PR, partial response; SD, stable disease; NR, no response; NRM, non-relapse mortality; yr, year; PFS, progression free survival; EFS, event free survival; LFS, leukemia free survival; DFS, disease free survival; OS, overall survival; FU, follow-up; MRD, minimal residual disease; aGvHD and cGvHD, acute and chronic graft versus host disease; BM, bone marrow; HA-1H is a hematopoiesis-restricted MiHA presented in HLA-A∗02:01; mbIL21, membrane-bound interleukin 21; NMC, non-myeloablative conditioning; MAC, myeloablative conditioning; DC, dendritic cells; rhIL-2, recombinant human interleukin-2; G-CSF, granulocyte colony-stimulating factor.

DLI has been shown to boost the GvL activity and to improve the patient outcome, especially in the setting of MRD and pre-emptive settings. However, the risk of subsequent GvHD is considerable and may increase the NRM, thus reducing the OS benefit of this cellular therapy.

### 3.3 Selected DLI

In order to reduce the GvHD risk and maintain a GvL effect, the DLI product can be manipulated. Back to 2004, Alyea and colleagues published a study using CD8^+^ depleted DLI for the treatment of disease recurrence after HCT. Patients were targeted to receive 3x10^7^ CD4^+^ T cells/kg. Nine patients were enrolled, 3 CML, 3 myeloma, 2 CLL, and 1 NHL. CD8 depletion was highly specific, with a median recovery of CD4^+^ cells of 75%. All CML patients achieved a complete molecular remission. A CLL patient demonstrated a complete response. One patient developed grade II acute GVHD and subsequently chronic GVHD (93). A subsequent study demonstrated that response to DLI was associated with quantity of preexisting marrow CD8^+^ T cells and local reversal of T-cell exhaustion (94).

Murine models showed that CD8^+^CD44^hi^ memory T (TM) cells could eradicate malignant cells without inducing GvHD (95). Muffly and colleagues evaluated the feasibility and safety of infusing freshly isolated and purified donor-derived CD8^+^ TM cells into patients relapsed after HCT. Fifteen patients received CD8^+^ TM cells at escalating doses, 9 had active disease and 13 received cytoreduction before cell infusion. No adverse infusion events or dose-limiting toxicities occurred. GvHD developed in 1 patient (grade 2 liver) and 10 patients (67%) maintained or achieved a response. Median EFS and OS were 4.9 months (1-19.3 months) and 19.6 months (5.6 months to not reached), respectively (96).

Regulatory T cells (Treg) are CD4^+^ T lymphocytes that express CD25 and FoxP3 and are negative for the CD127 marker. These cells play a crucial role in modulating the immune response and may reduce the GvL activity (97–99). Some groups have tried to eliminate these cells from the DLI product to boost the anti-tumor activity. Maury and colleagues treated 17 patients with relapses of various hematological malignancies after HCT. All patients had failed to respond to standard DLI and had no history of GvHD. Treg-depleted DLI were prepared by depleting CD25^+^ cells from donor leukapheresis using anti-CD25 magnetic microbeads. Compared with unmanipulated DLI, Treg-depleted DLI showed a better GvL effect, however with an increased GvHD incidence (6 out of 17 patients). With a mean follow-up of 6-years, Treg-depleted DLI treatment was associated with improved survival among the whole trial cohort (100). Colleagues from Dana-Farber Cancer Institute explored the use of Treg-depleted DLI in a phase I study treating 21 patients with different hematologic malignancies who had relapsed after transplantation. Seven subjects (33%) developed GvHD by 1 year, including one patient who died. The 1-year survival rate was 53% among patients treated with dose level 2. A shorter period between relapse and infusion was associated with response at dose level 2. Compared to unmodified DLI in 14 contemporaneous patients meeting study eligibility, Treg-depleted DLI treatment was associated with a better response rate and improved EFS (101).

The DLI product can also be manipulated to reduce the risk of GvHD, especially in the setting of mismatched donors. In a Phase II multicenter study of 23 patients with acute leukemia, a haploidentical, naïve T cells-enriched product, depleted of recipient-alloreactive T cells, called ATIR101 was infused in a single dose at 1 month after a T-cell depleted haploidentical HCT. Patients received a MAC conditioning with anti-thymocyte globulin (ATG) as a sole GvHD prophylaxis. NRM at 1-year was inferior for patients receiving ATIR101 concerning recipients of conventional haploidentical HCT, as well as improved GRFS (102). In another Phase I study, CD45RA^+^ naïve T-cells depleted DLI was given at 2 months following HLA-identical non-myeloablative HCT for hematologic malignancies. One patient developed grade 2 acute GvHD of skin and GI, and one moderate chronic GvHD of the lungs following the DLI. After a median follow-up of 2.8 years, 2-year PFS and OS were 50% and 68.8%, respectively (103). An ongoing phase I study (NCT03939585) is testing the safety of prophylactic TCR-αβ T and B cells depleted DLI infused on day 28 following HCT from MRD or haploidentical donor. This study hypothesizes that this DLI enriched in NK and TCR-γδ T cells displays a better GvL effect without increasing the risk of GvHD.

Finally, manufacturing T cells that can selectively target leukemia associated antigens (LAAs) represents an opportunity to promote antileukemic activity without inducing GvHD. Wilms tumor antigen 1 (WT1) is a transcription factor overexpressed in some leukemias. HLA-A*0201–restricted WT1-specific donor-derived CD8+cytotoxic T cell (CTL) clones were safely administered after HCT in 11 patients. Transferred cells exhibited direct evidence of antileukemic activity in two patients: a transient response in one patient with advanced progressive disease and the induction of prolonged remission in a patient with MRD. Additionally, 3 treated patients at high risk for relapse after HCT survived without leukemia relapse, GVHD, or additional anti-leukemic treatment. In this study, the exposure of CTLs to interleukin-21 (IL-21) increased *in vivo* CTL survival (104). Another strategy is to edit the TCR to be specific for a tumor antigen (105). However, TCR gene transfer results in competition for surface expression and inappropriate pairing between the exogenous and endogenous TCR chains, resulting in suboptimal activity and potentially harmful unpredicted specificities. To overcome this limitation, Bonini and colleagues designed zinc-finger nucleases (ZFNs) promoting the disruption of endogenous TCR β and α chain genes. ZFN-treated lymphocytes lacked CD3/TCR surface expression. These cells were subsequently infected with a lentiviral to express a WT1-specific TCR. these TCR-edited cells did not mediate off-target reactivity while maintaining anti-tumor activity *in vivo* (106).

Selected DLI have shown to reduce the risk of GvHD, while maintain the desirable GvL activity. However, the best approach to apply has not been defined yet.

### 3.4 Natural killer cell strategy: NK DLI and cytokine-induced memory-like NK cells

Several studies have shown a correlation between transplant outcomes and an impaired NK cell recovery, especially after haploidentical transplant with PTCy, suggesting the need to boost NK cells recovery early after transplant (47, 107–109). NK cells have long been utilized in the field of cellular therapy, leveraging their ability to recognize and kill tumor cells in a non-MHC-mediated manner (49). Moreover, they exhibit a better toxicity profile compared to T cells, reducing the risk of GvHD and the need for autologous sources, and opening the possibility of off-the-shelf, ready-to-use products. An important limitation of NK cell therapy is associated with their limited ability to persist *in vivo* and maintain immunologic memory (42, 110–112).

Various forms of NK cells have been tested in clinical settings, including haploidentical NK cells, cord blood-derived NK cells, stem cell-derived NK cells, NK-CAR cells, and CIML NK cells (113). Devillier and colleagues tested in a phase I study the prophylactic infusion of IL-2 activated NK cells after HCT from MRD with favorable results (114). Colleagues from MD Anderson employed an NK expansion method using K562 feeder cells expressing membrane-bound IL21 and 4-1BBL (FC21) to prepare NK DLI. In this phase I/II study, 25 patients received 3 prophylactic doses of donor NK cells administered on days −2, +7, and +28 after haploidentical HCT. After a median follow-up of 24 months, the 2-year relapse incidence and the LFS were improved compared to contemporaneous 160 case-match controls from the CIBMTR dataset (4% vs. 38%, and 66% vs. 44%, respectively). Only one relapse occurred in the study group, in a patient with a high level of donor-specific anti-HLA antibodies (DSA) presented before transplantation. Acute GvHD occurred in 10 patients and no patient developed chronic GvHD (115). Other trials testing the prophylactic NK cell infusions following HCT are ongoing (NCT03300492 and NCT02452697).

Interleukin 15 (IL-15) is a cytokine that stimulates both CD8^+^ T cell and NK cell antitumor responses. Romee and colleagues first tested this hypothesis in patients with myeloid malignancies relapsed after HCT using the IL-15 superagonist complex ALT-803. Thirty-three patients were treated with ALT-803 without observing any no dose-limiting toxicities or GvHD. Responses were observed in 19% of evaluable patients, including 1 complete remission lasting 7 months (116). The same group explored the use of recombinant human IL-15 (rhIL-15) after lymphodepleting chemotherapy and haploidentical NK cells. Of 26 treated patients, 36% had robust *in vivo* NK-cell expansion at day 14, and 32% achieved CR. The use of rhIL-15 was associated with cytokine release syndrome (CRS) in 56% of the cases and some cases of neurological toxicity (117). This study included some patients relapsed after a previous HCT.

CIML NK cells are generated ex vivo after cultivation with IL-12, IL-15, and IL-18, conferring a typical memory-like phenotype, an increased ability to secrete cytokines and exert cytotoxicity, and enhanced *in vivo* persistence (weeks/months) (118). Furthermore, they displayed excellent anti-leukemic activity both *in vitro* and *in vivo*. The first phase I human study (NCT01898793) showed the safety and initial efficacy of allogeneic CIML NK cells, along with concurrent administration of IL-2, in relapsed/refractory (R/R) AML patients (objective response rate - ORR 5/9 patients, including 4 complete responses) (119). These results were confirmed by a subsequent phase I study, demonstrating the safety and initial efficacy of CIML NK cells even in the context of relapse after HCT (120). This study reported the results of the first 6 treated patients, showing a rapid expansion of NK cells, lasting for several months. Fever and cytopenia were reported as the most common adverse events.

NK cell-based DLI have shown promising results, breaking down the GvHD incidence and enhancing the GvL effect. However, the poor persistence of the NK cells remains a major limitation of this approach, leading to an unsatisfactory duration of response and limited benefit in terms of patients OS.

### 3.5 Cytokine-induced killer cells

CIK cells represent a rare T cell subpopulation, recapitulating both T and NK cell features. CIK cells are obtained starting from peripheral blood mononuclear cells activated in culture in the presence of monoclonal antibodies against CD3 (OKT3), interferon-gamma (IFN-g), and interleukin-2 (IL-2) (121). IL‐2 and fresh culture medium need to be supplemented regularly until after 14-21 days of culture. At this point, an enrichment of CIK cells is observed, characterized by the expression of markers typical of NK cells and T cells, especially defined as CD3^+^, CD56^+^, with a prevalent CD8^+^ phenotype (122). Of note, CIK cells are a cluster of heterogeneous cells comprising CD3^+^CD56^−^ T cells, CD3^−^CD56^+^ NK cells, and CD3^+^CD56^+^ NKT cells (121, 123). CIK cells have the unique ability to recognize and kill malignant or infected cells, both through TCR-mediated cytotoxicity typical of T cells and through non-MHC restricted recognition, similar to NK cells, which is the so‐called “dual‐functional capability” (124–126). Indeed, as T cells, CIK cells express the polyclonal TCR repertoire. However, similarly to NK cells, they show an increased expression of cytotoxic receptors including NKG2D, DNAX accessory molecule-1 (DNAM-1), TRAIL, FasL, LFA-1, low density of NKp30 and CD16, and inducing the secretion of perforin and granzyme, but lack expression of NKp44, NKp46, KIR2DL1, KIR2DL2, KIR3DL1, NKG2A, CD94 (122, 127). Thanks to these characteristics, CIK cells have the unique ability to induce a GvL effect with a low risk of exacerbating GvHD (128–131).

In 2010 the International Registry on CIK cells (IRCC) was established, to collect and evaluate clinical trials using CIK cells for the treatment of different types of cancer. In the last update from the IRCC, a total of 106 clinical trials including 10,225 patients were collected, of which 4,889 patients in over 30 distinct tumor entities were treated with CIK cells alone or in combination with conventional or novel therapies. Of note, only 8 and 5 studies used allogeneic CIK cells and UCB-derived CIK cells, respectively. CIK dose varies between studies, with a wide range of 7.9 × 10^8^ to 7.9 × 10^10^. The number of infusions also varied according to the treatment efficiency or adverse effects. Ten studies reported that more cycles of CIK cell infusion were significantly related to the prolonged OS and PFS (132). In this meta-analysis including mostly solid tumor trials, the ORR was 38% with 9 studies reporting a significantly increased 5‐year survival rate. Moreover, mild adverse effects were observed and the rate of acute and chronic GvHD was modest and in most cases was controlled by the administration of immunosuppressive corticosteroids (133, 134). Initial studies using autologous CIK cells showed feasibility and no adverse events, but limited efficacy (135–137). Subsequent clinical trials tested allogeneic CIK cells in the setting of post-HCT relapse (138–148) Results of these trials are summarized in **Table 3**. Globally, these data confirmed the safe profile of CIK cells and the low rate of GvHD (not exciding 25% for aGvHD).

**Table 3:** Clinical trials on cytokine-induced killer (CIK) cell after HCT.

Study	Study design	Patients (N)	Disease (N of pts)	Setting	CIK source	Cell dose/N infusion	Additional treatment	Response	GvHD	Outcome
Introna et al. Haematologica 2007 (140)	Single- center, open-label phase I study	11	AML (4) HD (3) CML (1) ALL (1) MDS (2)	Treatment	MRD MUD	Median dose 12.4×10^6^ cells/kg (range 7.2-87.4) (median infusions 2 range, 1-7)	None	1 SD 1 PR 3 CR	4 aGvHD (grade I-II) 2 cGvHD	Alive 5/11
Introna et al. BBMT 2010 (139)	Compassionate use study	5	AML (4) ALL (1)	Treatment	UCB	A median of 1.5×10^6^ cells/kg (range: 1-8×10^6^ cells/kg (median infusions 1 range, 1-4)	CT	1 CR	1 aGvHD (grade III)	Alive 0/5
Laport et al. BBMT 2011 (142)	Single center open-label phase I study	18	NHL (n=5) AML (n=3) MM (n=3) CLL (n=2) ALL (n=2) MDS (n=2) HD (n=1)	Treatment	MRD	Escalation doses: 1×10^7^ CD3^+^ cells/kg 5×10^7^ CD3^+^ cells/kg 1×10^8^ CD3^+^ cells/kg (single infusion)	None	2 CR 8 disease related deaths	2 aGvHD (grade II) 1 limited cGvHD	Alive 10/18
Linn et al. BBMT 2012 (143)	Phase I/II clinical study	24 (16 infused)	AML (8) ALL (3) CML (1) HD (3) NHL (1)	Treatment	MRD (15) MUD (8) UCB (1)	from 10 to 200×10^6^ cells/kg (1-12 infusions)	None	5 ORR	3 aGvHD	2-years Alive in CR 2/16
Rettinger et al. BMT 2013 (147)	single- center study	2	AML	Treatment	Haplo (2)	15-170 x10^6^ cells/kg (from 7 to 9 infusions)	None	2 PR	None	2/2 dead
Rettinger et al. Haematologica 2016 (146)	Retrospective multicenter study	13	AML (5) ALL (7) CML (1)	Treatment	MRD (1) MUD (6) Haplo (6)	From 5 to 100 x10^6^ cells/kg (from 1 to 6 infusions)	None	10 CR 1 PR 2 NR	6 aGvHD (3 grade I and 3 grade III) 3 cGvHD	OS 69%
Luo et al. Leukemia Research 2016 (138)	single- center, open-label phase I study	15	AML (4) ALL (2) CML (3) MM (2) NHL (4)	Treatment	MRD (8) Haplo (7)	From 1×10^6^ to 8×10^7^ cells/kg (from 1 to 10 infusions)	HCT	15 ORR	2 aGvHD 1 grade I and I grade III	6 Alive
Introna et al. BBMT 2017 (141)	Multicenter, open-label phase IIA, study	74	AML (41) ALL (19) HD (3) MM (4) NHL (2) MDS(2) MPN (2)	Treatment	MRD (37) MUD (31) Haplo (5)	1×10^6^ cells/kg 5×10^6^ cells/kg 10×10^6^ cells/kg (3 infusions)	DLI	19 CR (26%) 3 PR (4%) 8 SD (11%)	12 aGvHD (16%), 7 of grade I-II 5 grade III-IV 11 cGvHD (15%)	3-year PFS 29% OS 40%
Pfeffermann et al. Cytotherapy 2018 (148)	Compassionate use study	1	PTLD	Treatment	MUD	10×10^6^ CIK cells/kg; 9.57×10^3^ EBV- specific CIK cells/kg	IL-15 and EBV peptide pool	1 CR	None	2-years alive in CR 1
Merker BBMT 2019 (145)	Retrospective, single- center, study	36 (32 pediatric)	AML (15) ALL (18) CML (1) NHL (2)	Treatment (8) Pre-emptive (17) Prophylaxis (11)	MRD (3) MUD (10) MMUD (23)	MRD/MUD median 10×10^6^/Kg (0.7-200) MMUD median 5×10^6^/Kg (0.1-9.4) (median infusions 2, range 1-9)	None	53% CR (68% CR pre-emptive+prophylactic)	9 aGvHD (25%) 8 grade I-II 1 grade III 2 limited cGvHD (6%)	0/8 alive (treatment) 6-months OS 77% (81% prophylaxis, 75%pre-emptive)
Narayan et al. BBMT 2019 (144)	Single- center, open-label phase II study	44 (31 infused)	AML (n=12) MDS (n=27) MPN (5)	Prophylaxis	MRD MUD MMUD	Escalation doses: 1×10^7^ cells/kg 3×10^7^ cells/kg 1×10^8^ cells/kg ^(^single infusion)	None	/	1-year aGvHD 25.1%	2-year NRM 6.8% EFS 27.3% OS 50.6%

N, number; pts, patients; yr, year; MRD, matched related donor; MUD, matched unrelated donor; MMUD, mismatched unrelated donor; Haplo, haploidentical donor; UCB, umbilical cord blood; ORR, overall response rate; PR, partial response; NR, no response; PFS, progression free survival; EFS, event free survival; OS, overall survival; FU, follow-up; NRM, non-relapse mortality; AML, acute myeloid leukemia; ALL, acute lymphoblastic leukemia; T-LBL, T-cell lymphoblastic lymphoma; MDS, myelodysplastic syndrome; MPD, myeloproliferative neoplasm; MM, multiple myeloma; NHL, non-Hodgkin lymphoma; HD, Hodgkin disease; CLL, chronic lymphocytic leukemia; CML, chronic myeloid leukemia; PTLD, post-transplant lymphoproliferative disorder; aGvHD and cGvHD, acute and chronic graft versus host disease; DLI, donor lymphocyte infusion.

Our group showed promising results generated by phase I studies demonstrating the feasibility and safety of clinical-grade allogeneic CIK cell production (139, 140). Moreover, we conducted a phase II study on the sequential use of DLI and CIK in 74 patients relapsed post-transplant, demonstrating a low incidence of GvHD and adequate disease control. Acute GvHD was observed in 16% of patients, of these, 7 presented with grade I-II and in 5 with grade III-IV. Chronic GvHD manifested in 15% of cases. A CR was observed in 26%, partial response (PR) in 4%, stable disease (SD) in 11%, and progression in 56% of cases. PFS at 1 and 3 years was 31% and 29%, respectively (141). Other groups have confirmed the low rates of acute and chronic GvHD after CIK infusion as a treatment for post-HCT relapse (142, 144, 145). In addition, a recent retrospective analysis from the Frankfurt group, comparing CIK cell infusions with DLI in a pediatric enriched cohort of HCT patients showed a low rate of acute and chronic GvHD and exciting outcomes when used in a prophylactic/pre-emptive setting (145). Of note, in this study, 63% of patients had a MMUD donor and CIK cells were produced by adding IL-15 and harvested after a shorter period (10-12 days of culture). In view of this low risk of alloreactivity, a phase I/II trial using haploidentical donor derived CIK cells for post-HCT relapse is currently ongoing at our institution (NCT03821519).

Finally, CIK cell therapy provides a suitable platform for combination strategies with monoclonal antibody (anti-CD20) (149), bispecific drugs, such as blinatumomab (anti-CD3 and anti-CD19) (150), immune checkpoint inhibitors including PD‐1, PD‐L1, KIR, LAG‐3, or TIM‐3 (151) or gene modification through the insertion of Chimeric Antigen Receptor (CAR) (152). The latter strategy has also been tested for AML (153–155). Also, some studies demonstrated that CIK cells stimulated by or armored with IL‐6, IL‐7, IL‐12, IL‐15, IL‐21 or thymoglobulin manifested phenotype alteration, proliferation improvement, and cytotoxicity enhancement (156–160).

Data from different trials conducted in various institutions worldwide have shown the ability of CIK cell infusions to boost the GvL activity, with high rate of response especially in the setting of MRD and pre-emptive therapy and with a minimum impact on GvHD onset, leading to an OS benefit.

## 4 Genetically engineered cell therapy strategies

### 4.1 Chimeric antigen receptor T cells

CAR-T cell therapy is an emerging novel therapeutic strategy in the context of relapsed/refractory lymphomas and leukemias, including AML (161). CARs have revolutionized the concept of immunotherapy by combining the specificity of monoclonal antibodies with the cytotoxic activity mediated by T-cells against tumor-associated antigens (TAA). The CAR consists of an extracellular antigen-binding domain, derived from the fusion of a variable portion of the light and heavy chain of immunoglobulins (scFv), and an intracellular portion characterized by one or more activating domains for T-cells, particularly the zeta chain of CD3 belonging to the T-cell receptor (TCR) complex (162). In the beginning, the approach for CAR-T cell development involved the use of viral vectors (adenovirus or lentivirus). However, the high production costs and complex manufacturing processes limited their clinical use. The introduction of non-viral transfections, using transposons through the Sleeping Beauty (SB) system, has proven to be an efficient and safe alternative (163, 164). Besides the choice of CAR construct design and the method of transgene insertion into the cell genome (viral or non-viral), the selection of the effector cell can have an impact on the treatment outcome.

Autologous CAR-T cell therapy has shown excellent results in the treatment of B-cell malignancies, such as B-cell acute lymphoblastic leukemia (B-ALL), B-cell non-Hodgkin lymphomas (B-NHL), chronic lymphocytic leukemia (CLL), and multiple myeloma (MM) (165–173). Since 2017, six different CAR-T cell therapies have been approved by the U.S. Food and Drug Administration (FDA) and the European Medicines Agency (EMA), including 4 anti-CD19 CAR-Ts and 2 anti-BCMA CAR-Ts for the treatment of B-ALL, B-NHL, and MM (174).

Differently, the use of allogeneic T cells poses unique challenges owing to potential alloreactivity. Allogeneic CAR-T cells can be collected either from an allogeneic donor (donor-derived) or from patients who relapsed after HCT (patient-derived). **Table 4** reported studies using either autologous or allogeneic CAR source as long as administrated after an HCT. Patient-derived donor T cells may be expected to carry a lesser risk of acute and chronic GVHD if the CAR T cells are generated from tolerized cells. Of note, data on patient-derived allogeneic CAR cells were collected from trials using mostly autologous CAR-T outside the setting of HCT. In these trials, the rate of GVHD was often not reported, but some of these studies show low rate or absence of GvHD after CAR-T cell therapy (166, 175–182). However, more recent studies did report some incidence of both aGvHD and cGvHD (183, 184). Unfortunately, the value of donor chimerism at the time of lymphocyte apheresis was nearly always not available.

**Table 4:** Clinical trials on chimeric antigen receptor (CAR) cell therapy after HCT.

Study	Study design	CAR product	Number of patients infused/enrolled (after HCT)	Disease	HCT Donor	CAR source	Response	GvHD post CAR-T	Outcome
Patient-derived T cell									
Grupp et al. NEJM 2013 (175)	Case report	Anti-CD19 4-1BB CAR-T; LV	1 (post HCT 68% donor chimerism)	B-ALL	UBC	Patient-derived	CR	None	CD19- relapse
Lee et al. Lancet 2015 (176)	Phase 1, dose-escalation study	Anti-CD19 CD28z CAR-T; RV	21/21 (8)	B-ALL	Not specified	Patient-derived	CR 50%	None	OS 51.6% LFS 78.8% (median FU 10 months)
Turtle et al. J Clin Invest 2016 (177)	Phase I, dose-escalation study	Anti-CD19 4-1BB CAR-T; LV; (CD4/CD8 = 1/1)	30/32 (11)	B-ALL	MRD (2) MUD (7) UBC (1) Haplo (1)	Patient-derived	11 CR	1 cGvHD	/
Gardner et al. Blood 2017 (178)	Phase I/II study	Anti-CD19 4-1BB CAR-T LV; (CD4/CD8 = 1/1)	45/45 (28)	B-ALL	Not specified	Patient-derived	CR 93%	1 aGvHD (grade III)	/
Jacoby et al. Am J Hem 2018 (242)	Phase Ib/II study	Anti-CD19 CD28z CAR-T	20/21 (10)	B-ALL	Not specified	Patient-derived	18 CR	None	1-yr EFS 73% OS 90% (all underwent HCT after CAR-T)
Maude et al. NEJM 2018 (179)	Phase I/II study	Anti-CD19 4-1BB CAR-T; LV	75/92 (46)	B-ALL	Not specified	Patient-derived	23 CR 31 CRi	None	6-months EFS 67%, OS 78%
Park et al. NEJM 2018 (166)	Phase I study	Anti-CD19 CD28z CAR-T; RV	53/83 (19)	B-ALL (19)	Not specified	Patient-derived	CR 83%	None	median EFS 6.1 months OS 12.9 months
Abramson et al. The Lancet 2020 (243)	Phase I multicenter study	Anti-CD19 4-1BB CAR-T (LisoCel); LV	269/344 (9)	NHL	Not specified	Patient-derived	ORR 73%	None	/
Zhang et al. Blood Advances 2020 (183)	phase I/II study	Anti-CD19 4-1BB or CD28z CAR-T; LV	110/115 (16)	B-ALL	MRD, MUD, Haplo	Patient-derived (14) Donor-derived (2)	15 CR	2 aGvHD (grade I and grade III) 2 cGvHD	1-year LFS 30.5% OS 30.5%
Shah et al. Blood 2021 (244)	Phase I/II study	KTE-X19 (Brexu-cel) anti-CD19 CD28z CAR-T; RV	45/54 (13)	B-ALL	Not specified	Patient-derived	ORR 69% CR 53% CRi 16%	None	Median DOR 14.5 months
Shah et al. JCO 2021 (245)	Phase I dose-escalation, single-center study	Anti-CD19 CD28z CAR-T; RV	50/53 (22)	B-ALL	Not specified	Patient-derived	CR 62%	Not reported	Median OS 10.5 and EFS 3.1 months
Shah et al. Lancet 2021 (180)	Phase, multicenter, single-arm, open-label study	KTE-X19 (Brexu-cel) anti-CD19 CD28z CAR-T; RV	55/71 (24)	B-ALL	Not specified	Patient-derived	CR/CRi 71%	None	Median DOR 12,8 RFS 11,6 OS 18,2 months
Shah et al. Immunotherapy Cancer 2023 (246)	Long follow-up ZUMA-3 study	KTE-X19 (Brexu-cel) anti-CD19 CD28z CAR-T; RV	78/X (38)	B-ALL	Not specified	Patient-derived	/	None	Median DOR with (n=14) and without (n=43) subsequent HCT was 44.2 and 18.6 months
Ghorashian et al. Blood 2024 (218)	Phase I, open label study	Anti-CD19 CAR-T and CD22 CAR-T; LV	12/13 (6)	B- ALL	Not specified	Patient-derived	10/12 CR (83%)	Not reported	OS 75% EFS 60% at 12-month
Liu et al. Am J Hematol. 2021 (184)	Phase I study	Anti-CD19 CAR-T and CD22 4-1BB CAR-T; LV	27/32 (27)	B- ALL	MUD (6) Haplo (20) UBC (1)	Patient-derived	23 CR 3 PR 1 early death	2 aGvHD (grade II) 4 cGvHD	18-months EFS 65% OS 84%
Berdeja et al. Lancet 2021 (247) and Martin et al. JCO 2023 (248)	Phase Ib/II multicenter study	Anti-BCMA 4-1BB CAR-T (Cilta-cel); LV	101/113 (8)	MM	Not specified	Patient-derived	ORR 97.9%	Not reported	1-yr PFS 77% 1-yr OS 89%
Cowan et al. Lancet Onco 2023 (249)	First in human phase I study	Anti-BCMA CAR-T; LV + crenigacestat (LY3039478)	18/19 (2)	MM	Not specified	Patient-derived	ORR 89%	Not reported	Median PFS 11 months OS 42 months
Jin et al. J Hematol Oncol. 2022 (250)	First in human phase I study	Anti-CLL1 4-1BB CAR-T; LV	10/10 (5)	AML	Not specified	Patient-derived	CRi 70%	Not reported	6 patients alive at the end of the last FU
Zhang et al. Leukemia. 2022 (251)	Phase I/II study	Anti-CLL-1 CD28z-CD27 CAR-T; LV	8/8 (2)	AML	Not specified	Patient-derived	5 CR 1 CRi 1 PR 1 SD	Not reported	/
Naik et al. Blood 2022 (252)	Phase I study	Anti-CD123 CD28z, CD20 CAR-T; LV	6/13 (11)	AML	Not specified	Patient-derived	2 CR 1 PR	Not reported	/
Voorhees et al. Blood Advances 2020 (182)	Case report	Anti-CD30 CAR-T	1 (with previous HCT, with 100% chimerism)	EATL	MRD	Patient-derived	CR	None	30 months in CR
Tambaro et al., Leukemia 2021 (181)	Phase I study	Anti-CD33 4-1BB CAR-T; LV	3/10 (3)	AML	Not specified	Patient-derived	No response	None	Died for disease progression
Donor-derived and patient-derived T cell									
Budde et al. Blood 2017 (189)	First in human phase I study	Anti-CD123 CD28z CAR-T; LV	7/14 (6)	AML (6) BPDCN (1)	Not specified	Donor-derived Patient-derived	3 ORR	1 GvHD	/
Cui et al. J. Hematol Oncol 2021 (253)	Phase I/II study	Anti-CD38 CD28 4-1BB CAR-T	6/6 (6)	AML	MUD (2) Haplo (4)	Donor-derived (2) Patient-derived (4)	4 CR/CRi	None	/
Lu et al. Blood 2022 (254)	Phase I study	Naturally selected anti-CD7 4-1BB CAR-T (NS7CAR); LV	20/20 (5)	T- ALL (14) T-LBL (6)	Not specified	Donor-derived (2) Patient-derived (18)	5 CR 14 CRi 1 NR	aGvHD 1 (grade I)	4/6 patients who did not receive a HCT remained in CR
Donor-derived T cell									
Cruz et al. Blood 2013 (187)	Phase I study	Anti-CD19 CD28z CAR-T; RV	8/8 (8)	B-ALL (4); CLL (4)	MRD (5) MUD (2) MMUD (1)	Donor-derived	2 CR	None	1 remained in CR for 8 months and the other 1 for 8 weeks
Kochenderfer et al. Blood 2013 (188)	Phase I study	Anti-CD19 CD28z CAR-T; RV	10/10 (10)	CLL (4) DLBCL (2) MCL (4)	MRD (6) MUD (4)	Donor-derived	CR (1) PR (1) SD (6)	None	/
Dai et al. Oncoimmunology 2015 (255)	Pilot study	Anti-CD19 4-1BB CAR-T; LV	9/9 (3)	B-ALL	Not specified	Donor-derived (2) Patient-derived (1)	1 CR	2 aGvHD	/
Brudno et al. JCO 2016 (185)	Phase I dose escalation study	Anti-CD19 CD28z CAR-T; RV	20/20 (20)	B-ALL (5) CLL (5) DLBCL (5) MCL (5)	MRD (13) MUD (6) MMUD (1)	Donor-derived	6 CR 2 PR	2 cGvHD	/
Kebriaei et al. JCI 2016 (186)	Phase I dose escalation study	Anti-CD19 CD28z CAR-T; SB	26/50 (19)	B-ALL (17) NHL (2)	MRD (10) Haplo (9)	Donor derived	11 CR	2 aGvHD 1 cGvHD	1-yr PFS 53% OS 63%
Jia et al. Journal of Hematology & Oncology 2019 (191)	Case report	Anti-CD19 and CD19/CD22 bispecific 4-1BB CAR-T	1 (after HCT)	B-ALL	Haplo	Donor-derived	CR	1 aGVHD (grade IV)	/
Zhang et al. Leukemia 2021 (190)	Phase II study	Anti-CD19 4-1BB or CD28 CAR-T; LV	43/43 (43)	B-ALL	MRD (17) Haplo (26)	Donor-derived	CR 79%	2 GvHD (grade I-II)	1-year EFS and OS 43% 1-year CIR 41%
Yang et al. Blood Cancer Journal 2022 (256)	First-in-human single-arm, single-center, proof-of-concept phase I study	Anti-CD19 CD28 F-CAR-T; LV	25/25 (1)	B-ALL	Not specified	Donor-derived	CR 92%	None	15 pts were disease-free with a median DOR 734 days (20/23 proceed to HCT)
Li et al. Transplantation and cellular therapy 2023 (217)	Phase I study donor CD7 CAR-T therapy followed by allo-HSCT from the same donor	Anti-CD7 4-1BB; LV	12/12 (1)	T- ALL (10)	MRD 1	Donor-derived	CR 91%	3 aGvHD (2 grade II 1 grade IV) 3 cGvHD	1-year OS 92% and DFS 57%
Donor-derived non-T cell									
Magnani et al. JCI 2020 (152) and Lussana et al. Blood 2022 (202)	Phase I/II study	Anti-CD19 CD28z OX-40 CARCIK; SB	27/27 (27)	B-ALL	MRD (7) MUD (10) Haplo (10)	Donor-derived	CR 66.7%	None	6-months EFS 41.5% and OS 71.4%; median DOR 9.5 months
Marin et al. Nat Med 2024 (193) and Liu et al. NEJM 2020 (192)	Phase I/II study	Anti-CD19-CD28z-iCasp9-IL15 CAR-NK; RV	37/41 (1)	B-ALL (1) NHL (25) CLL (11)	UBC	Donor-derived	day 100 OR rates were 48.6%	None	1-yr PFS 32% OS 68%
Tang et al. Am J Cancer Res 2018 (207)	Phase I, first-in-man study	Anti-CD33 CD28z and 4-1BB CAR NK cells; LV	3/3 (1)	AML	NK-92 cell line	Cell-derived	0	Not reported	/
Huang et al. Hemasphere 2023 (208)	Phase I	Anti-CD33 CAR NK	10/10 (not reported)	AML	Not reported	Donor-derived	6 CR (MRD^-^)	Not reported	/
Modified donor-derived T cells									
Sallman et al. Blood 2022 (194)	Phase I, open-label, dose-escalation multicenter study	UCART123v1.2 Anti-CD123 4-1BB CAR-T, (TALEN editing TCRa^ko^ CD52^ko^)	17/17 (9)	AML	non-HLA–matched healthy donor cells	Donor-derived	ORR 4/17	0	1 persisting CR MRD^-^
Jain et al. Blood 2020 (195) and Boissel et al. Hemasphere 2023 (257)	Phase I open-label dose-escalation study	UCART22 (TALEN editing TCRa^ko^ CD52^ko^)	18/19 (8)	B-ALL	non-HLA–matched healthy donor cells	Donor-derived	ORR 7/18	/	/
Benjamin et al. Lancet Haema 2022 (196)	Phase 1, open-label, multicenter study	UCART19 anti-CD19 4-1BB CAR-T; LV (TALEN editing TCRa^ko^ CD52^ko^)	25/25 (18)	B-ALL	non-HLA–matched healthy donor cells	Donor-derived	ORR 48%	2 aGvHD (grade I) (8%)	DOR 7,4 PFS 2,1 OS 13,4 months Median FU 12,8 months
PAN et al. JCO 2021 (197) and Tan et al. Journal of Hematology & Oncology 2023 (258)	Phase I study	Anti-CD7 4-1BB CAR-T; LV; KDEL	20/20 (12)	T ALL	MRD 5 MUD 1 Haplo 14	Donor-derived	ORR 95% CR 90%	aGvHD 60% (11 grade I and 1 grade II) 1 late onset aGVHD grade IV; 7 cGvHD	2-yrs PFS 36.8% and OS 42.3% Median PFS 11.0 and OS 18.3 months
Hu et al. Cell Res 2022 (198)	Phase I study	RD13-01 anti-CD7 4-1BB CAR-T; RV (CRISPR-Cas9 editing HLA-II^ko^CD7^ko^ TRC^ko^NKi^+^)	12/12 (2)	T ALL (11) AML (1)	non-HLA–matched healthy donor cells	Donor-derived	CRi 4; CR 3; PR 2	None	/
Ottaviano Sci Tran Med 2022 (199)	Phase 1, open-label, study	TT52CAR19 anti-CD19 CAR-T; LV (CRISPR-Cas9 editing TCRa^ko^ CD52^ko^)	6/8 (5)	B-ALL	non-HLA–matched healthy donor cells	Donor-derived	CR 66%	1 aGvHD (grade I)	/
Chiesa et al. NEJM 2023 (200)	Phase I study	(BE-CAR7s) anti-CD7 4-1BB CAR-T; LV (BE CD52^ko^CD7^ko^ TRCb^ko^)	3/3 (2)	T ALL	non-HLA–matched healthy donor cells	Donor-derived	2 CR MRD^-^ 1 CRi MRD^+^	None	/
Modified patient-derived T cells									
Cummins at al. Blood 2017 (259)	Pilot open-label study	Biodegradable T cells anti-CD123 4-1BB CAR mRNA tandem TCR	6/7 (4)	AML	Not specified	Patient-derived	No response	Not reported	0
Wermke et al. Blood 2021 (214) and Blood 2023 (215)	Phase I dose-escalating study	UNICART, anti-TM CD28z + TM123	19/19 (12)	AML	Not specified	Patient-derived	ORR 53% 75% (for MRD^+^)	Not reported	Median DOR 5 months

N, number; MRD, matched related donor; MUD, matched unrelated donor; MMUD, mismatched unrelated donor; haplo, haploidentical donor; LV, lentivirus; RV, retrovirus; SB, sleeping beauty; Flu, fludarabine; Cy, cyclophosphamide; MM, multiple myeloma; ORR, overall response rate; DOR, duration of response; yr, year; PFS, progression free survival; EFS, event free survival; LFS, leukemia free survival; DFS, disease free survival; OS, overall survival; FU, follow-up; AML, acute myeloid leukemia; ALL, acute lymphoblastic leukemia; T-LBL, T-cell lymphoblastic lymphoma; NHL, non-Hodgkin lymphoma; EALT, enteropathy-associated T cell lymphoma; TM, target molecule; MRD, minimal residual disease; KDEL, endoplasmic reticulum retention signal sequence; aGvHD and cGvHD, acute and chronic graft versus host disease; BM, bone marrow; CCR, continuous complete remission; CR2, second CR; DIR, died in remission; LV, lentiviral transduction; PR, partial remission; gRL, g-retroviral transduction; SB, Sleeping Beauty transposon; BE, base editing; F-CAR-T, fast CAR-T; NS7CAR, naturally selected CD7 CAR-T.

Surprisingly, initial reports using donor-derived allogeneic CAR-T cells showed a low incidence of GvHD. These studies, however, did not include lymphodepleting regimens and showed limited efficacy (185–188) More recent trials have tested allogeneic CAR-T cells with lymphodepletion in the setting of disease recurrence after HCT (189, 190) and even haploidentical HCT (184, 191) with positive results.

However, several strategies are under investigation to mitigate the risk of causing or aggravating GvHD. One possibility is to choose different effector cells other than T cells, such as CIK cells (152) or NK cells (192, 193). Another possibility is to perform gene editing to delete the endogenous TCR (194–200). Interestingly, with the exemption of two studies (196, 197), no GvHD was reported in this series of patients. Finally, also the CAR-T cell production method plays a role in improving treatment efficacy. The goal is to favor protocols that promote the differentiation of T cells into stem memory cells (SCM) or central memory T cells (CM), which have shown better anti-tumor activity compared to effector memory T cells (EM) (201).

CAR cell therapy has shown unprecedented results in different hematologic diseases, however data on the use of allogeneic products is still limited.

### 4.2 Alternative effector cell sources: genetically modified CIK and NK cells

CIK cells can be employed as alternative effectors for CAR cell therapy. Anti-CD19 CAR-CIK cells have shown an excellent safety profile and good response rates in patients with post-HCT R/R B-ALL (152, 202). In this study, no GVHD following CAR-CIK was reported. Preclinical studies using CAR-CIK cells against CD33 and CD123 have shown promising results also in the setting of AML (153–155). CAR-CIK cells were also tested *in vitro* against CD44v6 with encouraging results (203). In addition, one case report form China, showed a transient response in an AML patient treated with autologous CAR-CIK cells direct against CD33 (204). Finally, preclinical data showed that CAR cell activity can be enhanced by increasing BM homing through the expression of specific chemokines (CXCR4) (205) or by armoring them to constitutively secrete cytotoxic cytokine (IL-18) (206).

CAR-NK cells can be generated from either NK tumor lines (NK92), induced pluripotent stem cells (iPSCs) differentiated into NK cells, or isolated NK cells from peripheral or UCB (113). UCB-derived CAR-NK cells demonstrated impressive clinical efficacy in CD19-positive diseases (192, 193). In this study, only one patient presented a post-HCT relapse/refractory disease and no case of GvHD was reported. NK92-derived CAR-NK cells, targeting CD33 and constitutively secreting IL-2, were used to treat 3 patients with relapsed/refractory AML. This study observed no toxicity but also no efficacy (207). NK cells derived from NK92 do not express some activating/cytotoxic receptors such as CD16 and NKp44, representing a limitation of this approach. Huang and colleagues presented preliminary data from a phase I study on multiple infusions of allogeneic CAR-NK cells targeting CD33 in 10 patients with relapsed/refractory AML, reporting no toxicity, and 6 out of 10 patients achieved MRD-negative complete remission by day 28 (208). Allogeneic CAR-NK cells targeting CD123, derived from the peripheral blood of healthy donors, demonstrating anti-leukemic activity *in vitro* and *in vivo* without toxicity to the normal healthy myeloid compartment (209). Finally, Dong and colleagues proposed an innovative approach, where NK cells were induced to differentiate into CIML-like NK cells and then transduced with a CAR recognizing the neopeptide derived from cytosolic oncogenic nucleophosmin-1 (NPM1c), presented by HLA-A2 (210). Currently, several clinical trials are recruiting patients with relapsed/refractory AML for treatment with CAR-NK cells targeting different epitopes: CD123, CD33, CCL1, and NKG2DL (211).

Both allogeneic donor-derived CAR-CIK and CAR-NK cell strategies have shown to exert a potent GvL activity with a minimum GvL toxicity. Long-term survival analysis and data on CAR cell persistence are needed to select the best strategy.

### 4.3 Novel strategy to improve CAR-T cell therapy

One goal for the improvement of cellular therapy with CAR-T cells is the use of fit, ready to use donor T cells, without exacerbating the risk of GvHD. Gene editing can be used to manipulate the T cell, by deleting the endogenous TCR, allowing the use of allogeneic non matched or third part T cells. Most studies on T-cell editing initially began by deleting a TCR gene, then introducing a CAR using a retroviral or lentiviral vector (212).

UCART are gene-edited CAR-T cells engineered using a TALEN editing strategy, to knock down the TCR and the CD52 receptor. TALENs are chimeric proteins that contain two functional domains: a DNA-recognition transcription activator-like effector (TALE) and a nuclease domain. This technology allows the use of third-party T cells, tearing down the risk of GvHD and the use of alemtuzumab within the lymphodepleting regimen without the risk of CAR-T depletion. Indeed in 3 different studies only 2 cases of grade I aGvHD were reported (194–196).

In the setting of anti-CD7 CAR T cells, different editing strategies were used to block the expression of CD7, the T cell receptor (TCR), and human leukocyte antigen (HLA) class II, to avoid CAR T cell fratricide, GvHD, and rejection, respectively (197–200). Furthermore, in the study of Hu and colleagues, the expression of the NK inhibitor (NKi) and the common cytokine receptor gamma chain (gc) was induced to enhance CAR-T cell cytotoxic activity (198).

However, an interesting mouse study from the Memorial Sloan Kettering Cancer Center showed that allogeneic second generation CD28 CAR-T cells have less GvHD capacity while maintaining an anti-lymphoma capability compared to first generation or second generation 4-1BB CAR-T cells (213). These data suggested a natural tolerogenic profile of CD28 CAR-T cells, while maintaining their endogenous TCR.

Another approach to reduced CAR-T cell toxicity involves the “universal” CAR-T cells (UniCAR). This second-generation autologous CAR construct using a CD28 costimulatory domain is designed to bind a soluble target module (TM) able to bind the tumor antigen (CD123). In this phase I study, 19 patients were treated, of which 12 after a relapse post-HCT. The ORR was 53% and no case of GvHD was reposted (214, 215). Interestingly, treatment-related toxicities quickly resolved with the suspension of the target module administration.

Finally, to overcome the selection of the therapeutic target considering the shared expression of antigens by both myeloid precursors and malignant cells, valid alternatives include post-CAR-T cell HCT (216, 217), targeting AML specific antigens (such as NPM1) (210), and the use of dual-targeting CAR-T cells capable of recognizing more than one target and fully activated only when both antigens are expressed on the target cell (155). In addition, by targeting multiple antigens, the dual-CAR construct can potentially overcome the tumor immune escape arising from antigen-negative disease, such as in the case of CD19 loss (218).

Although the real risk of GvHD after donor-derived CAR-T cells is still not well defined yet, novel gene editing strategies can be effectively employed to mitigate the T cell alloreactivity in the context of allogeneic or third-part CAR-T cell therapy.

## 5 Conclusions

Despite the successful rate of cure after HCT, a considerable fraction of patients still experiences disease recurrence or persistence. CIK cell-based therapy represents a suitable approach to boost the GvL activity without increasing GvHD. Indeed, CIK cells offer an allogeneic cell platform with a tolerogenic profile and can be considered also for a third-part ready to use cellular therapy. In addition, novel strategy using CAR-CIK cells showed efficacy with limited toxicity and no report of GvHD in a setting of heavily pretreated patients. Major limitations are represented by the limited efficacy in the setting of overt hematological relapse and lack of suitable targets and severe toxicities in the myeloid setting. Moreover, high-level evidence coming from phase III randomized controlled trials (RCTs) is needed in this field. Future perspective includes the use of prophylactic CIK cell infusion, the use of novel modified CIK cells, such as armored-CIK cells or CAR-CIK cells for the treatment of disease recurrence after HCT.
